# Hydrogen-Bond Restructuring of Water-in-Salt Electrolyte Confined in Ti_3_C_2_T_*x*_ MXene Monitored by Operando Infrared Spectroscopy

**DOI:** 10.1021/acs.jpclett.2c03769

**Published:** 2023-02-07

**Authors:** Mailis Lounasvuori, Tyler S. Mathis, Yury Gogotsi, Tristan Petit

**Affiliations:** † Nanoscale Solid−Liquid Interfaces, Helmholtz-Zentrum Berlin für Materialien und Energie GmbH, 14109 Berlin, Germany; ‡ Department of Materials Science and Engineering and A. J. Drexel Nanomaterials Institute, Drexel University, Philadelphia, Pennsylvania 19104, United States

## Abstract

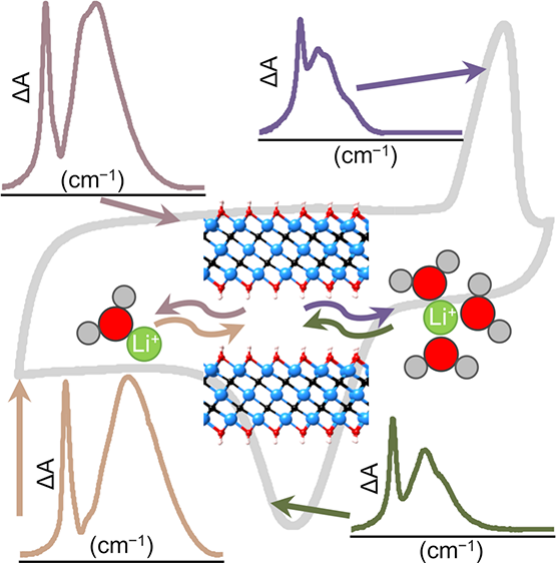

Highly concentrated water-in-salt aqueous electrolytes exhibit a wider potential window compared to conventional, dilute aqueous electrolytes. Coupled with MXenes, a family of two-dimensional transition metal carbides and nitrides with impressive charge storage capabilities, water-in-salt electrolytes present a potential candidate to replace flammable and toxic organic solvents in electrochemical energy storage devices. A new charge storage mechanism was recently discovered during electrochemical cycling of Ti_3_C_2_T_*x*_ MXene electrodes in lithium-based water-in-salt electrolytes, attributed to intercalation and deintercalation of solvated Li^+^ ions at anodic potentials. Nevertheless, direct evidence of the state of Li^+^ solvation during cycling is still missing. Here, we investigate the hydrogen bonding of water intercalated between MXene layers during electrochemical cycling in a water-in-salt electrolyte with operando infrared spectroscopy. The hydrogen-bonding state of the confined water was found to change significantly as a function of potential and the concentration of Li^+^ ions in the interlayer space. This study provides fundamentally new insights into the electrolyte structural changes while intercalating Li^+^ in the MXene interlayer space.

Li-ion batteries outperform other battery technologies in many respects and are therefore the technology of choice for smartphones, laptops, and other portable digital devices.^1^ However, in order to incorporate intermittent renewable energy into electrical grids and to achieve widespread vehicle electrification, the development of large-scale electrochemical energy storage solutions is needed.^2^ Beyond the scarcity of lithium, the current technology relies on flammable organic solvents and stringent, water-free processing and operating conditions.^3^ Aqueous electrolytes are a safer and greener alternative to organic solvents but suffer from a narrow potential window (<1.2 V) limited by the decomposition of water, thereby excluding the use of many conventional Li-ion electrochemical couples.^4^ However, increasing the concentration of the salt in an aqueous electrolyte expands the potential window by two mechanisms: First, the thermodynamic reaction potential of oxygen evolution increases due to the lower activity of water.^5^ Second, in stark contrast to conventional aqueous electrolytes, the hydrogen evolution potential at the anode is reduced by the formation of a solid-electrolyte interphase.^6^ The widespread use of water-in-salt electrolytes (WiSEs) is hampered by the high viscosity of concentrated electrolytes.^7^ It is therefore important to understand the critical physicochemical phenomena that suppress the hydrogen evolution reaction (HER) and enable the extended electrochemical stability window of WiSEs. The unique electrochemical properties of WiSEs may be reproducible at lower electrolyte concentrations with improved understanding of the nanoscale phenomena occurring at the electrode–electrolyte interface in WiSE systems.

MXenes are of particular interest for use in aqueous electrolytes containing halogen ions, as they are stable in chloride, bromide, and other solutions and, due to high metallic conductivity, do not require metal current collectors that may corrode in salt solutions.^8^ Whereas in conventional dilute aqueous electrolytes, such as 1–3 M sulfuric acid, Ti_3_C_2_T_*x*_ MXene is oxidized at anodic potentials beyond 0.1–0.2 V vs Ag/AgCl,^9,10^ the use of highly concentrated electrolytes protects MXene from oxidation and hydrolysis^8^ while also extending the potential window toward more cathodic potentials.^11−13^ So far, studies of MXene in such electrolytes, have focused on ion transport^12^ or electrochemical performance.^8,14^ Kim et al.^12^ used electrochemical impedance spectroscopy to elucidate the energy barriers for ion transport in the LiTFSI electrolyte at different concentrations. Avireddy et al.^13^ reported an asymmetric MXene-MnO_2_ supercapacitor with excellent rate capability and low self-discharge in potassium acetate WiSE. Increased pseudocapacitive Li^+^ intercalation was observed in LiBr WiSE when the Ti_3_C_2_ MXene was partially oxidized.^14^ In addition, the use of WiSE has been proposed to activate a new electrochemical process involving desolvation-free intercalation of cations, contributing to additional capacitance.^8^ This novel process shows dramatic interlayer spacing changes with a higher number of water molecules (de)intercalating with Li^+^ at a substantially different insertion potential, resulting in distinct peaks in the current. The interlayer spacing at positive potentials after the oxidation peak was found to be ca. 1.6 Å, less than the diameter of a water molecule and consistent with 0.33 H_2_O molecules and zero Li^+^ ions in the interlayer space per unit cell, as determined by DFT calculations.^8^ At negative potentials after the reduction peak, the interlayer spacing jumped to 3.5 Å, best described by a structure with 1.08 H_2_O and 0.25 Li^+^ per Ti_3_C_2_^8^ and in close agreement with the optimized structure of one water layer in the Ti_3_C_2_ interlayer space.^15^ It can be distinguished from the typical pseudocapacitive cation intercalation in MXenes observed in dilute aqueous electrolytes, where Li^+^ intercalates in a partially desolvated state and the cyclic voltammogram displays a nearly rectangular shape.^16^

In the above-mentioned work by Wang et al.,^8^ the nature of the Li^+^ solvation shell was only extracted from indirect measurements (gravimetric, interlayer spacing, and DFT), while the anomalous electrochemical process suggests that the cation solvation structure may be very different compared to the dilute electrolyte. Moreover, since the hydrogen evolution reaction depends strongly on the number and strength of hydrogen bonds (H-bonds) in which the reactant water molecules participate,^17,18^ it is important to characterize the water H-bonding environment in a concentrated electrolyte where HER is suppressed.

Operando methods allow the study of processes at electrode surfaces to probe structural (X-ray diffraction, electrochemical atomic force microscopy) or chemical (X-ray absorption, Raman spectroscopy) changes in electrode materials upon cycling. However, very few techniques are sensitive to the electrolyte itself. Attenuated total reflection Fourier transform infrared spectroscopy (ATR-FTIR) fills this gap. The water O–H stretch is particularly sensitive to the H-bonding environment of the water molecule and can therefore be used to study the structure of the solvation shell around the intercalating cation in the MXene interlayer space. In addition, ATR-FTIR is an accessible method with simple sample preparation and versatile electrochemical cell configuration, offering the possibility to study dynamic processes under relatively realistic conditions.

Here, we used operando ATR-FTIR spectroscopy to study the vibrational signature of confined water in Ti_3_C_2_T_*x*_ MXene during electrochemical cycling at potentials covering both desolvation-free and partially solvated Li^+^ (de)intercalation processes. We observed distinct spectral features as a function of potential and the H_2_O/Li^+^ ratio within the interlayer space and correlated the change in the water stretching mode with the intercalation mechanism.

The experimental setup is presented schematically in Figure 1A. In operando measurements, FTIR spectra are acquired simultaneously during cyclic voltammetry, without stopping the potential at any fixed value. Each spectrum was averaged over a scanning range of 60 mV. The spectra were measured in the ATR mode, which helps to remove much of the bulk electrolyte signal if the thickness of the Ti_3_C_2_T_*x*_ film is greater than the probing depth (see SI for more details). The characterization of Ti_3_C_2_T_*x*_ MXene used in this study can be found in our earlier publication.^19^

**Figure 1:**
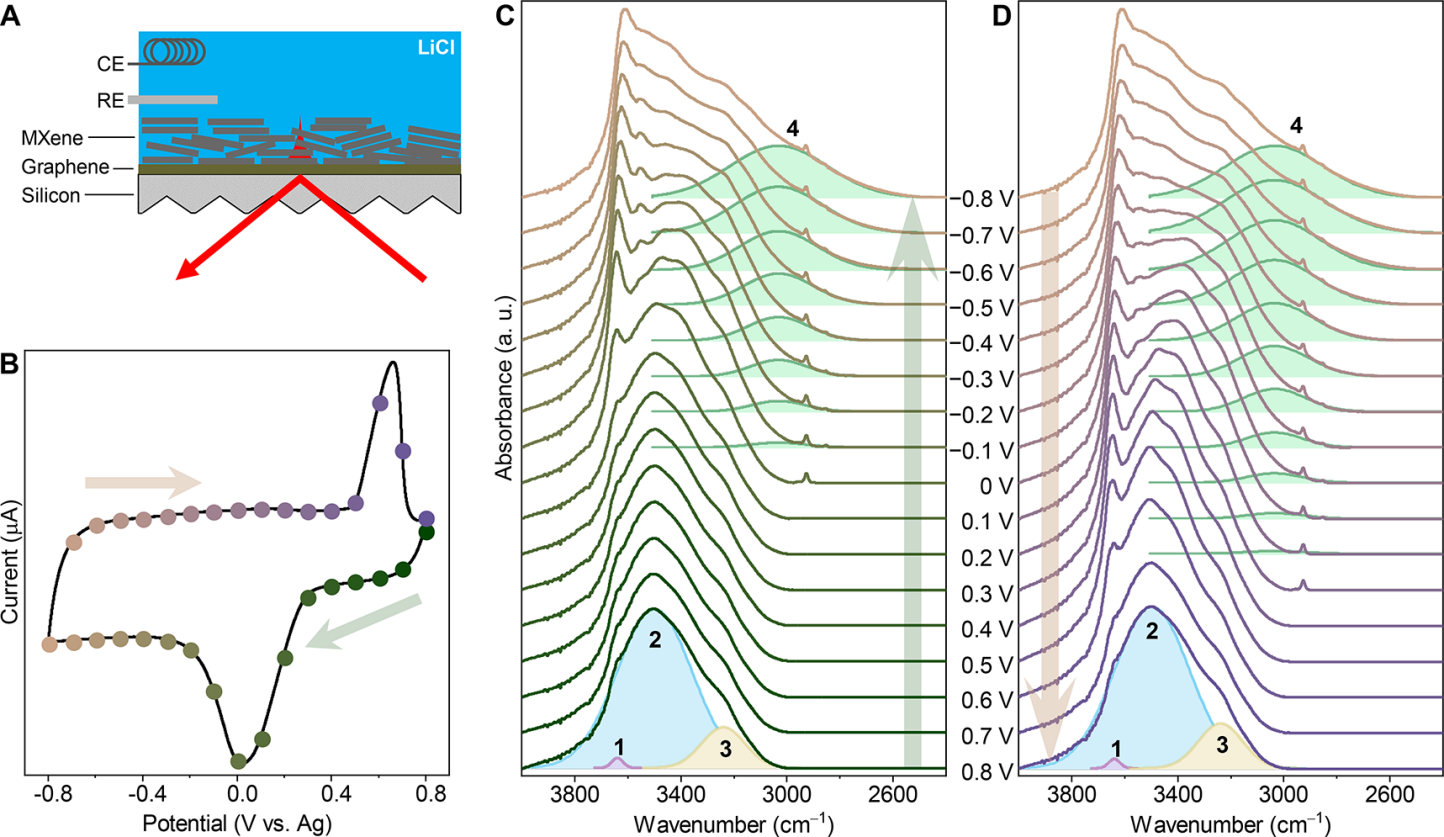
Operando FTIR of water stretching modes upon cycling in a Li^+^-based WiSE. (A) Schematic of the experimental setup. The counter and reference electrodes are labeled CE and RE, respectively. The Ti_3_C_2_T_*x*_ MXene film acts as the working electrode. (B) Cyclic voltammogram recorded during the operando FTIR measurement in 19.8 M LiCl. (C, D) Operando FTIR spectra of Ti_3_C_2_T_*x*_ MXene in 19.8 m LiCl recorded during (C) intercalation from +0.8 V to −0.8 V (bottom to top) and (D) deintercalation from −0.8 to 0.8 V (top to bottom). Arrows indicate scan direction. The four main components of the O–H stretch region are labeled 1–4.

The cyclic voltammogram (CV) recorded during the operando infrared measurement is presented in Figure 1B. Starting from +0.8 V, capacitive current is observed until the potential reaches +0.3 V, after which increased reduction current occurs, resulting in a reduction peak close to 0 V. After the reduction peak, large capacitive current is again seen during the rest of the cathodic sweep to −0.8 V and during the anodic sweep until +0.5 V, where the oxidation current begins to increase and an oxidation peak is observed close to +0.6 V. The CV is fully consistent with previously reported electrochemical data^8^ with only minor differences in peak potentials, which can be explained by differences in electrode preparation and electrochemical cell design. Extensive electrochemical characterization by Wang et al.^8^ shows that, despite their large separation, the peaks do not correspond to a battery-like, diffusion-limited Faradaic process but rather a surface-controlled charge storage process. The first cycle is shown in Figure S1.

Figure 1C shows the operando FTIR spectra of the water stretching modes during intercalation from +0.8 V to −0.8 V. At + 0.8 V; most Li^+^ has been expelled from the interlayer slits due to the high positive potential. The spectrum therefore corresponds to pure intercalated water and can be fitted with three main components corresponding to free O–H at ca. 3650 cm^–1^ (peak 1), weakly H-bonded water at ca. 3500 cm^–1^ (peak 2), and strongly H-bonded water at 3240 cm^–1^ (peak 3). Two additional minor components were included in the full fit presented in Figure 2. This spectrum strongly differs from bulk water (Figure S2), showing weaker H-bonding in intercalated water due to the strong 2D confinement in the interlayer space. Note that the IR spectral evolution observed in the MXene interlayer is overall very distinct from saturated LiCl solutions (Figure S3), which we attribute to the absence of anion contribution to the spectral features. Anions are repelled by the strong negative charge of Ti_3_C_2_T_*x*_, and only cations are intercalated into the space between MXene sheets.^20^ The integrated area of the water bending mode (Figure S4) increases in tandem with the stretching mode area. This indicates that the main contribution to the spectrum comes from intercalated water rather than any dissociated water that is surface-bonded to the MXene.^21^ During intercalation, the shape and intensity of the O–H stretch region remain essentially unchanged going from +0.8 V all the way to the reduction peak close to 0 V. After the reduction peak, a fourth component (peak 4) emerges, representing very strongly H-bonded water as indicated by the low frequency of 3030 cm^–1^. This component increases in area during Li^+^ intercalation until the negative vertex potential, at which point the scan direction is reversed. Operando spectra during the reverse scan are presented in Figure 1D. As the potential is scanned in the positive direction from −0.8 V, Li^+^ begins to deintercalate, and the area of peak 4 decreases until it vanishes at potentials above +0.3 V. The CV and infrared spectra are reproducible apart from a small increase in the signal from bulk-like water (Figure S5) after each cycle.

**Figure 2:**
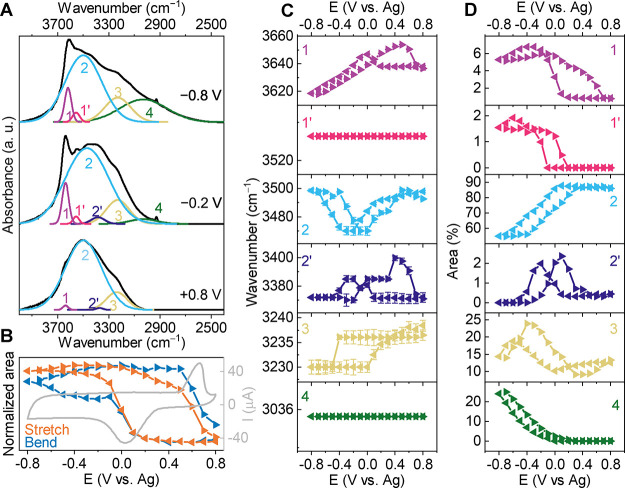
Evolution of water stretching modes upon cycling in the 19.8 m LiCl. (A) Peak fits of the initial spectrum at 0.8 V, after the reduction peak at −0.2 V, and at a negative vertex potential of −0.8 V. (B) Integrated, normalized total areas of stretching and bending modes as a function of potential; CV recorded during the operando measurement. (C) Frequencies and (D) integrated peak areas as a percentage of the total stretching area as a function of potential. Left-pointing and right-pointing triangles represent cathodic and anodic scan directions, respectively.

A more detailed look at the evolution of the fitted peaks during cycling provides further insights into the changes of the hydration shell of intercalated Li^+^. Peak fitting of the stretching mode region is shown for selected spectra in Figure 2A, corresponding to three critical potentials along the CV. The spectrum at +0.8 V represents the lowest number of Li^+^ ions in the Ti_3_C_2_T_*x*_ interlayer space. The spectrum at −0.2 V is recorded right after the reduction peak when the desolvation-free intercalation of Li^+^ has finished, and the spectrum at −0.8 V (the negative vertex potential) represents the highest number of Li^+^ ions within the Ti_3_C_2_T_*x*_ film. The total integrated areas of the stretching and bending modes are presented in Figure 2B. Figure 2C,D show how the frequencies and areas (as percentage of the total area) of the individual components of the stretching mode region evolve during the CV. The clear differences between the spectra obtained at these different potentials can be described in terms of changes of the water H-bonding resulting from Li^+^ (de)insertion as discussed in the following. The two minor components 1′ and 2′ observed in Figure 2A are omitted from the discussion due to their small areas, which never exceed 3% of the total area of the stretching mode region (Figure 2D).

An increase in the water H-bonding strength is known to cause a decrease in the frequency of the internal O–H stretch of a water molecule. The high frequency and narrow shape of peak 1 (Figure 2A) indicate that it arises from free O–H stretching modes.^22^ The H-bonding strength of the O–H stretches increases in the order peak 1 < peak 2 < peak 3 < peak 4 (Figure 2A). Fermi resonance of the bending overtone and the symmetric O–H stretch likely also contributes to peak 3.^23^ The frequency (3000 cm^–1^) and intensity of peak 4 indicate that, at negative potentials with a large amount of intercalated Li^+^, a considerable fraction of the water molecules in the interlayer space (25% of the overall stretching band) experience very strong H-bonding. Note that in a dilute electrolyte (0.1 M LiCl), this component represents less than 3% of the total stretching band (Figure S7), showing that it is a characteristic feature of WiSE at negative potential. Such a low frequency for the water O–H stretch, attributed to low-density water,^24^ has been predicted for confined water at high pressure.^25^ At high Li^+^ concentration, intercalated water molecules therefore experience a peculiar H-bonding environment similar to supercooled or high-pressure conditions.

The normalized total areas of the stretching and bending mode regions are plotted in Figure 2B (for spectra in the bending mode region, see Figure S4). The areas are not a quantitative measure of the amount of water within the interlayer spacing due to (i) the intensity of the O–H vibration changing in the presence of cation–water and water–water interactions^26^ and (ii) the signal originating from both intercalated water and water residing in larger mesopores present in the Ti_3_C_2_T_*x*_ film. Despite these limitations, a clear correlation with the CV is observed in the form of a sharp increase in both the stretching and the bending mode area coinciding with the oxidation peak. Correspondingly, a sharp decrease in the areas occurs during the reduction peak. A direct correlation is also seen between the area from the FTIR measurements and the expected amount of water entering/exiting the MXene film as determined by electrochemical quartz crystal microbalance (EQCM) measurements and density functional theory (DFT) calculations.^8^

The insertion/extraction of Li^+^ and H_2_O into/from the Ti_3_C_2_T_*x*_ electrode and the related changes in the water H-bonding are even more visible when considering the data as difference spectra (Figure 3). By subtracting the first spectrum recorded at +0.8 V from subsequent spectra, any contribution from bulk-like water present in the film is removed and the potential-induced changes in the intercalated H_2_O molecules are highlighted. Three different regimes (electrical double layer capacitance (EDLC) only, desolvation-free intercalation of Li^+^, and intercalation of partially desolvated Li^+^) are highlighted in Figure 3, shown next to the current curve. At high positive potential, only EDLC is observed. The intercalation of fully desolvated Li^+^ is rather unlikely, as we see very little change in the stretching modes of intercalated water in the IR spectra. At the reduction peak, desolvation-free Li^+^ intercalation occurs. The amount of water in the interlayer space increases rapidly, leading to an increase in the absorbance of components 1 and 2 of the OH stretching modes (Figure 2B). The difference spectrum at −0.2 V is most likely dominated by water molecules in the first solvation shell of Li^+^ only. After the reduction peak, Li^+^ intercalates in a partially desolvated state, and the number of water molecules per Li^+^ decreases. As a result, stronger H-bonding between intercalated water molecules is observed until −0.8 V, as evidenced by the progressive shift of the stretching mode maximum toward lower wavenumbers (Figure 3) due to the increase of the relative contribution of peaks 3 and 4 (Figure 2B). Stronger H-bonding has been shown to reduce the activity of water molecules and lead to suppressed HER.^17,18^ The strong H-bonding experienced by the intercalated water molecules in Ti_3_C_2_T_*x*_ at negative potentials is expected to have a similar effect, and therefore the local structure of the intercalated water is expected to contribute to the suppression of HER in a concentrated electrolyte. It is remarkable that at −0.8 V, most newly intercalated water molecules either form very strong H-bonds (peak 4) with either other water molecules or the MXene surface or are not H-bonded (peak 1). After the negative vertex potential, Li^+^ deintercalates in a partially desolvated state, leading to a rise in the number of H_2_O per Li^+^ and consequently a progressive weakening of H-bonding. At the oxidation peak, Li^+^ deintercalates in a desolvation-free state. The amount of water in the interlayer space decreases rapidly, leading to a decrease in the absorbance of the water stretching mode. The final state then becomes very close to a flat line, showing the good reversibility of the cycle.

**Figure 3:**
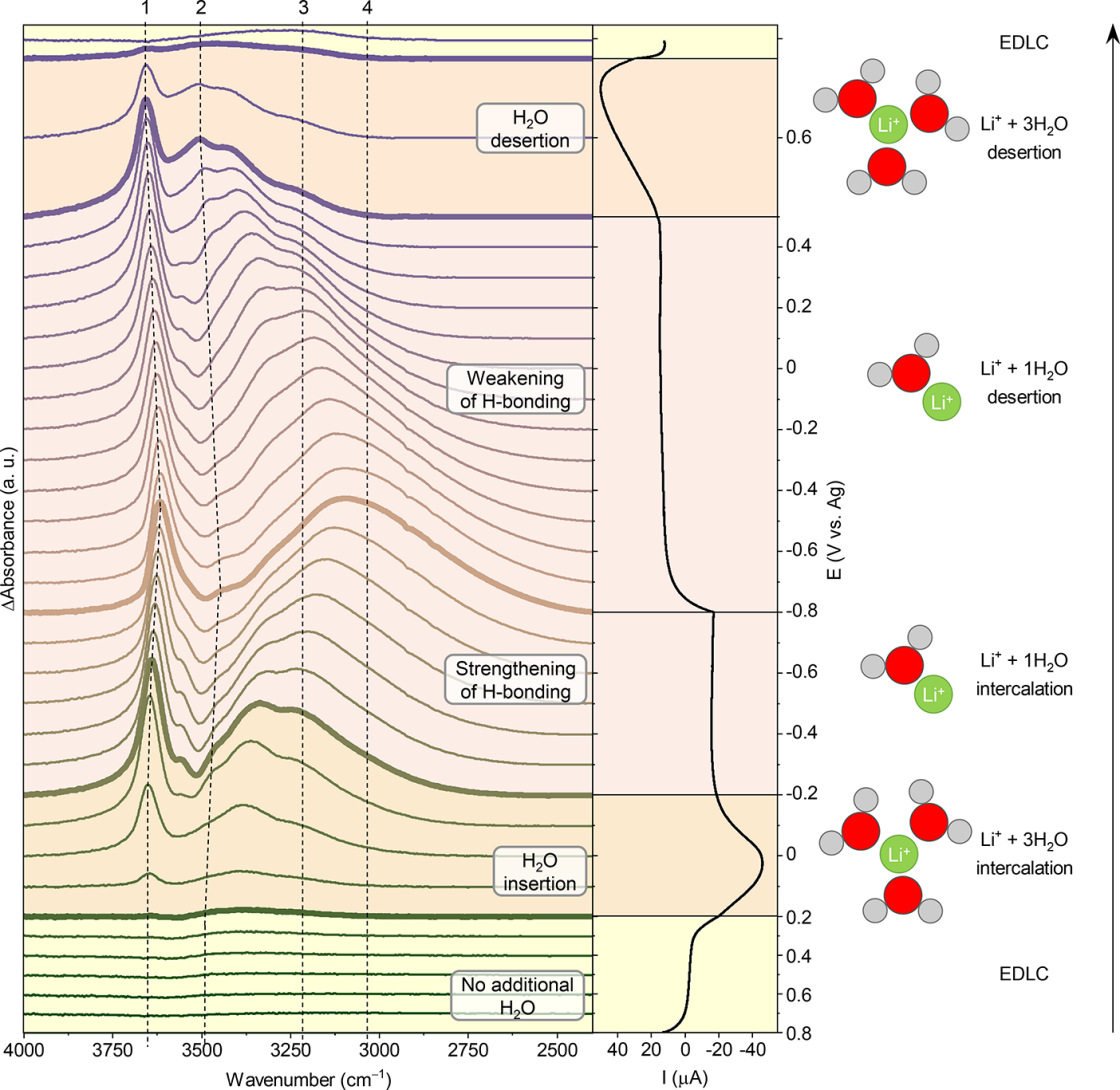
OH stretching modes of water molecules intercalated between the MXene planes in the 19.8 m LiCl. Difference spectra are obtained by subtracting the spectrum recorded at +0.8 V from the subsequent potentials. The current flowing through the MXene film is also shown as a function of potential. The three different intercalation regimes are highlighted in yellow, orange, and rose, and the respective solvation structure of intercalated Li^+^ species is schematically shown. For the EDLC regime, no Li^+^ is intercalated. The positions of the four main peaks obtained from deconvolution of the full spectra are indicated by dotted lines.

We therefore observed dramatic changes of the H-bonding of water molecules during cycling. The water being intercalated along Li^+^ ions presents predominant contributions from non-H-bonded water (peak 1) and strongly H-bonded water (peaks 3 and 4), whereas peak 2, a constituent of weakly H-bonded water molecules in bulk liquid water, barely contributes to the difference spectra. This can be explained by the fact that small cations with a high charge density affect water molecules in two distinct ways: on the one hand, water molecules in the hydration shell of Li^+^ are predicted to make fewer hydrogen bonds compared to bulk water;^27^ on the other, they form stronger donated H-bonds compared to bulk water due to Li^+^-induced polarization.^26^ In addition, confinement of the electrolyte enhances the contribution from loosely bonded water due to spatial constraints, as observed previously in carbon nanotubes.^28^ A slight decrease of the frequency of peak 1 is clearly visible for increasingly negative potential, which may be a result of increased ordering of water molecules. Similarly, a redshift of components 1–3 is seen in dilute LiCl at negative potentials (Figure S7). The small area of peak 4 in dilute electrolyte is most likely due to the much smaller number of Li^+^ intercalating into the MXene electrode compared to WiSE (see SI for more details). All in all, the water molecules intercalated in WiSE at negative potential are mostly contained in the first solvation shell of Li^+^ and experience strong reorganization upon further addition of Li^+^ in a partially desolvated state. These results point to a significant influence of the water H-bonding on electrochemical energy storage in confined environments, as was predicted by Sugahara et al.,^29^ and a similar effect of electrolyte reorganization has been observed in ionic liquids.^30^ Based on these observations, the formation of very strong H-bonds in the MXene interlayer space at high cation concentrations is a key process that leads to suppressing the HER. WiSE engineering strategies for MXenes should therefore focus on forming localized, strong H-bonds within the MXene interlayer space, a strategy that was reported previously for another aqueous battery system.^31^ Generating localized strong H-bonding may be possible by using strategies other than simply increasing salt concentration in the future. Promising strategies for future studies are preintercalation, the removal of nonstructural water, introducing electrolyte additives that strongly bond with water, or tuning of the surface chemistry of MXenes.

In this study, the structure of water confined in Ti_3_C_2_T_*x*_ MXene interlayer spaces during electrochemical cycling in a water-in-salt electrolyte was characterized using operando infrared spectroscopy. Two different lithium insertion charging mechanisms are operative at high Li^+^ concentration: the typical capacitive charging mechanism that takes place at cathodic potentials, also in a dilute electrolyte where lithium is inserted in a partially dehydrated form, and a desolvation-free charging mechanism that occurs at anodic potentials and is characterized by distinct redox peaks. Pronounced changes were observed in the vibrational signature of the MXene-confined water depending on the electrode potential and closely correlating with the two charging mechanisms. The IR spectrum of the lithium hydration shell confined within Ti_3_C_2_T_*x*_ interlayers shows significantly increased ordering of water, with the appearance of a strongly H-bonded water component, as well as a considerable number of free O–H bonds, evidenced by an intense, sharp band at 3650 cm^–1^. Strongly H-bonded water at negative potentials will have reduced activity, leading to suppressed HER. Ti_3_C_2_T_*x*_ MXene, a two-dimensional material, provides a unique opportunity to observe water molecules in 2D confinement and in the lithium hydration shell in the absence of anions or contact-ion pairs, and at a higher concentration of lithium than is achievable in bulk due to limits of solubility.

## Experimental Methods

### MXene Synthesis

Ti_3_C_2_T_*x*_ MXene was synthesized according to the procedure reported previously.^19^ By including excess aluminum during synthesis of the Ti_3_AlC_2_ MAX phase precursor, single- and few-layer Ti_3_C_2_Tx MXene was obtained with improved stoichiometry, resistance to oxidation, and increased electrical conductivity. Briefly, the MAX phase was synthesized from TiC, Ti, and Al powders at 1380 °C under a constant argon flow. The washed, dried, and sieved Ti_3_AlC_2_ precursor was etched in a mixture of HCl and HF to produce multilayered MXene, which was then delaminated by dispersing in a LiCl solution to obtain single- and few-layer Ti_3_C_2_T_*x*_ MXene. A stock aqueous suspension at a concentration of 5.9 mg/mL was stored under argon in a sealed bottle, and fresh aliquots were drawn on the day of the experiment.

### MXene Film Preparation

All electrolytes and Ti_3_C_2_T_*x*_ suspensions were made with doubly deionized water (Millipore, resistivity 18.2 MΩ·cm). LiCl (Sigma-Aldrich) was used as received. All electrolytes were deoxygenated by bubbling with nitrogen for 30 min prior to experiments. The internal reflection element (IRE) was a microstructured Si wafer (Irubis) covered by a monolayer film of graphene (Graphenea) transferred as per instructions. A new graphene-covered wafer was prepared for each measurement. The graphene layer improved adhesion between the Ti_3_C_2_T_*x*_ film and the IRE, leading to a higher sensitivity to interlayered water layers and in more reversible spectra during electrochemical cycling. A Ti_3_C_2_T_*x*_ MXene stock suspension was diluted to 1 mg/mL. A total of 125 μL was pipetted onto a graphene-covered Si wafer, and the droplet was allowed to dry at room temperature. Some manual manipulation of the drying droplet was necessary to ensure that the IR beam probed an area fully covered by Ti_3_C_2_T_*x*_. The resulting film was ca. 600 nm thick.

### Electrochemical Measurements

Electrochemical measurements were performed in a three-electrode spectroelectrochemical cell, designed and built in-house. Electrical contact with the Ti_3_C_2_T_*x*_ MXene sample working electrode was made through the graphene film, although measurements were also performed without the graphene layer with very similar electrochemical results. A coiled Pt wire and a Ag wire were used as the counter and quasi-reference electrodes, respectively. The potential was controlled with a Bio-Logic SP-200 potentiostat running EC-Lab software. With all electrodes mounted, the cell was filled with electrolytes, sealed, and mounted into the IR spectrometer, and the spectrometer was evacuated until the pressure in the sample chamber reached 0.8 mbar (typically ca. 1 h). After this equilibration time, the open-circuit potential (OCP) of the system was recorded and used as the starting potential for the measurement. Each CV was recorded between 0.8 V and −0.8 V with a scan rate of 2 mV/s. Several cycles were performed to verify the stability of the Ti_3_C_2_T_*x*_ electrode.

### FTIR Measurements

The measurements were carried out at the IRIS beamline at the BESSY II electron storage ring operated by the Helmholtz-Zentrum Berlin für Materialen und Energie.^32^ A conventional internal broadband IR source was used for the measurements. Infrared spectra were collected in the attenuated total reflectance (ATR) mode with a Bruker 70v spectrometer running Opus software. The optical accessory was designed and built in-house to accommodate the microstructured Si wafer as the IRE and provided an angle of incidence of 29°. Each spectrum consisted of 128 scans and took approximately 30 s to record, with a 20-s waiting time between spectra, resulting in one spectrum every 50 s. With a cyclic voltammetry scan rate of 2 mV/s, this equates to one spectrum every 0.1 V, with each spectrum being an average of 60 mV. The timing was manually synchronized with the cyclic voltammetry such that the midpoint of each spectrum collection coincided with potential = 0.1*n* V, where *n* = integer. For example, an IR spectrum, the collection of which started when the potential was −0.17 V and finished when the potential was −0.23 V, is designated as the spectrum at −0.2 V. A linear baseline was subtracted from absorbance spectra where necessary. No ATR correction was performed on the spectra. Some CH_*x*_ contamination resulting from the graphene transfer process is visible in the FTIR spectra, but this is judged to not have an effect on the potential-dependent changes observed.

## Supporting Information Available

Additional experimental details; additional FTIR spectra; details on peak fitting of FTIR data (PDF)
