# Supplementary material for: Hydrogen-Bond Restructuring of Water-in-Salt Electrolyte Confined in Ti3C2Tx MXene Monitored by Operando Infrared Spectroscopy
Source: J Phys Chem Lett. 2023 Feb 7;14(6):1578–84. doi: 10.1021/acs.jpclett.2c03769 (PMC9940289; doi:10.1021/acs.jpclett.2c03769)
Supplement: Supplementary file 1 — jz2c03769_si_001.pdf [file jz2c03769_si_001.pdf]

Hydrogen-Bond Restructuring of Water-in-Salt Electrolyte Confined in $Ti_3C_2T_x$ MXene Monitored by *Operando* Infrared Spectroscopy

SUPPORTING INFORMATION

Mailis Lounasvuori[1], Tyler S. Mathis[2], Yury Gogotsi[2], Tristan Petit[1*]

[1]Nanoscale Solid-Liquid Interfaces, Helmholtz-Zentrum Berlin für Materialien und Energie GmbH, 14109 Berlin, Germany

[2]Department of Materials Science and Engineering and A. J. Drexel Nanomaterials Institute, Drexel University, Philadelphia, PA, 19104, United States

Corresponding Author

*tristan.petit@helmholtz-berlin.de

Probing depth: In order to calculate the probing depth, we need to know the optical constants of $Ti_3C_2T_x$ MXene. However, these have not yet been experimentally determined in the mid-infrared range. Theoretical work on $Ti_3C_2(OH)_2$ in vacuum (1) predicts that the refractive index n varies between 2.5 and 4.9, and the extinction coefficient k is as high as 2, but the presence of voids in the film as well as intercalated water and ions will hugely impact the optical properties of the film. Based on the theoretical value, the ATR condition is not met using a Si ATR element and 28.74° angle of incidence. Work is ongoing to measure the optical constants experimentally so a model can be developed that allows us to calculate the penetration depth and absorbance spectra. We have previously investigated the penetration depth experimentally by observing the absorption bands from a piece of tape glued onto $Ti_3C_2T_x$ films of varying thicknesses (unpublished data). Since the *operando* measurements described here were performed with films ca. 600 nm thick, we can be sure that the probing depth in these measurements does not extend beyond the MXene film and that we are only probing water present inside the film, either confined between the MXene layers or existing in larger mesopores within the film.

Water-in-salt definition: Water-in-salt is defined as a salt solution where the weight or volume ratio of salt to water exceeds 1. (2) Strictly speaking our 19.8 m (mol kg^{-1}) LiCl solution does not fulfil this definition, but at such high concentration the ratio of $H_2O:Li^+$ is 2.8, far below the hydration number of 4-5 H_2O molecules per Li^+ in dilute solutions. (3)

First electrochemical cycle: In the first cycle, starting from OCP and scanning first to more negative potential, only capacitive current is observed (Figure S1). This capacitive regime continues during de-intercalation until the potential reaches ca. +0.5 V, when an anodic peak occurs. As the potential is scanned towards negative potential again, the cathodic peak also emerges. This shows that the expulsion of (nearly) all of the intercalated Li and water must occur first before desolvation-free Li^+ intercalation can take place.

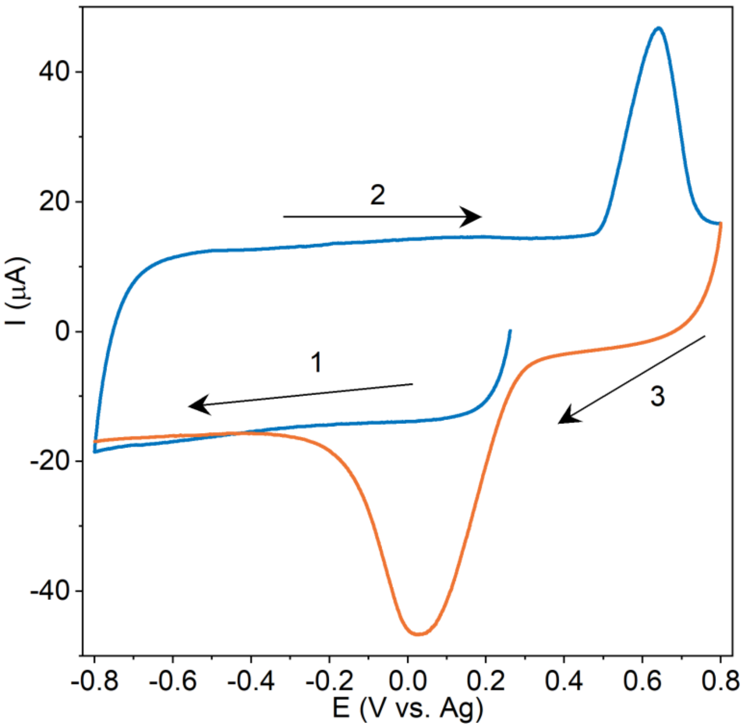

Figure S1. The first potential cycle of $Ti_3C_2T_x$ in 19.8 m LiCl. (1) The potential is first scanned from OCP in the negative direction. No reduction peak is observed in the blue trace. (2) After the negative vertex potential the scan is reversed. When the potential reaches ca. 0.5 V, an oxidation peak is observed. (3) After the positive vertex potential a reduction peak emerges at ca. 0.1 V (orange trace).

The FTIR signature of water confined between the $Ti_3C_2T_x$ MXene significantly differs from the bulk water measured in similar conditions as shown in Figure S2. In particular, a shift to larger wavenumbers, associated to a weakening of the H-bonding in the confined water, is clearly observed.

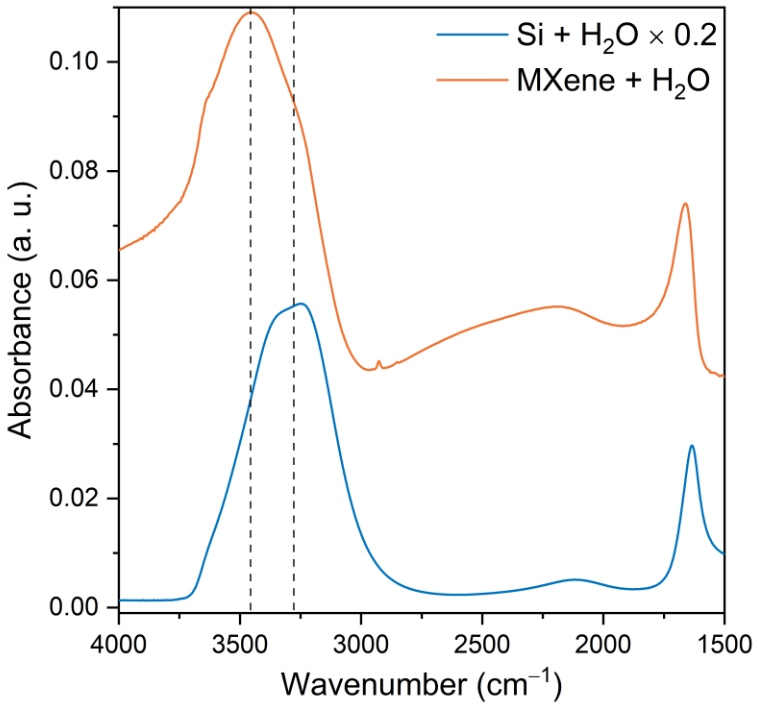

Figure S2. Effect of confinement on water spectrum. The bulk water spectrum (blue) was scaled by 0.2 and the confined water spectrum (orange) was stacked for clarity. Dashed vertical lines show the center of the O-H stretching mode band.

FTIR spectra of LiCl solutions: IR measurements of different concentrations of LiCl on bare Si and in contact with a $Ti_3C_2T_x$ MXene film are presented in Figure S3. For LiCl solutions on bare Si (Figure S3A,C), a clear change in the water stretching modes, related to a change of hydrogen bonding, is observed. This is a result of the progressive decrease in the Li^+ hydration number from 4 to 3. (4)

When the LiCl solutions are in contact with MXene (Figure S3B, D, F), the infrared signal originates from intercalated Li and water molecules within the MXene film rather than the bulk solution, so concentrations may be different to the nominal concentration. It has been established that anions do not spontaneously intercalate into the MXene interlayer spaces; (5, 6) we can therefore assume that we are characterizing the lithium hydration shell only in the absence of Cl^-. Normalized spectra in the O-H stretching mode and water bending mode regions are shown in Figure S3A, B, E, F. In comparison to the bulk spectra, a clearer shift to higher (stretch) and lower (bend) wavenumber is observed in the confined case, indicative of weaker hydrogen-bonding, as well as a more distinct narrowing of the bending mode also associated with weaker H-bonding as observed to occur with increasing temperature. (7) Difference spectra (with pure water spectrum subtracted) in Figure S3C, D show that in the bulk there is a negative band at 3640 cm^{-1} assigned to loss of free O-H bonds due to water interacting with anions. (8, 9) In the confined case (Figure S3D), there is no change at this wavenumber until the LiCl reaches a very high concentration, at which point a distinct peak appears

at 3645 cm^{-1}. This is assigned to the increased number of free O-H bonds due to excess cations disrupting the water H-bonding network and supports the assumption that we are characterizing the cation hydration sheath without the interference of anions.

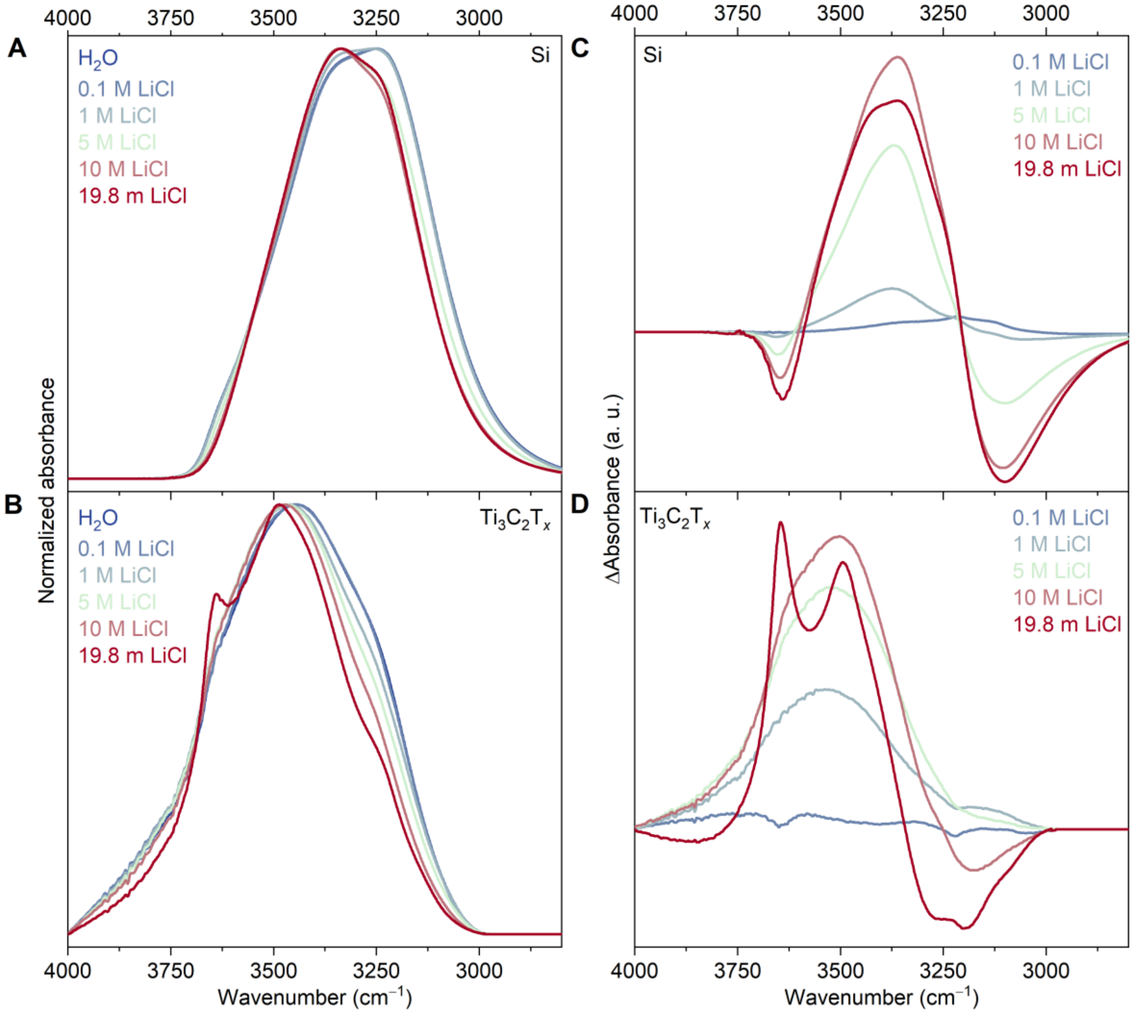

Figure S3. IR spectra of LiCl solutions. (A), (B): Normalized spectra in the O-H stretching mode region; (C), (D): Difference spectra in the O-H stretching mode region with pure water spectrum subtracted. (A), (C) (top row) were measured on bare Si; (B), (D) (bottom row) confined within $Ti_3C_2T_x$ MXene.

Peak fitting: The peak fit was performed in Igor Pro using Gaussian peak shapes. The fit was based on several previous reports on peak fitting of the O-H stretching region measured by Raman (10, 11) and infrared (12, 13) spectroscopy while considering components clearly visible in the spectra. A second derivative analysis was also used to support the fit. The fit is not intended to be quantitative, but rather a qualitative aid for describing the H-bonding state of the intercalated water molecules. The two smallest components were labelled 1' and 2' due to their proximity to main components 1 and 2. The inclusion of 1' improved the fit at negative potentials but this component was not apparent at potentials above ca. 0 V, whereas 2' was only included at intermediate potentials. The frequencies of components 1', 3 and 4 had to be constrained to achieve a meaningful fit; all other parameters were unconstrained. At this stage, the origin of components 1' and 2' remains to be clarified.

FTIR spectra of H_2O bending mode: The *operando* FTIR spectra were recorded from 6000 cm^{-1} to 750 cm^{-1}, thus covering simultaneously both the stretching and bending modes of water. The main manuscript focuses on the wavenumber region pertaining to the stretching mode. The bending mode of water is less strongly affected by the H-bonding state than the stretching mode (14) and has been used previously to quantify confined water in clay materials. (15) It therefore offers a potentially more reliable marker than the stretching mode for the amount of water in the $Ti_3C_2T_x$ interlayer spaces. Stronger H-bonding results in a blueshift of the bending mode in contrast to a redshift in the stretching mode, and a relationship between the stretching mode and bending mode frequency has been established. (16) The *operando* spectra in the bending mode region are presented in Figure S4. Even though we see greatly increased intensity at the lower frequency range of the stretching mode region, peak 1 remains the largest contributor to the overall area. It is therefore no surprise that the bending mode shifts very little in frequency.

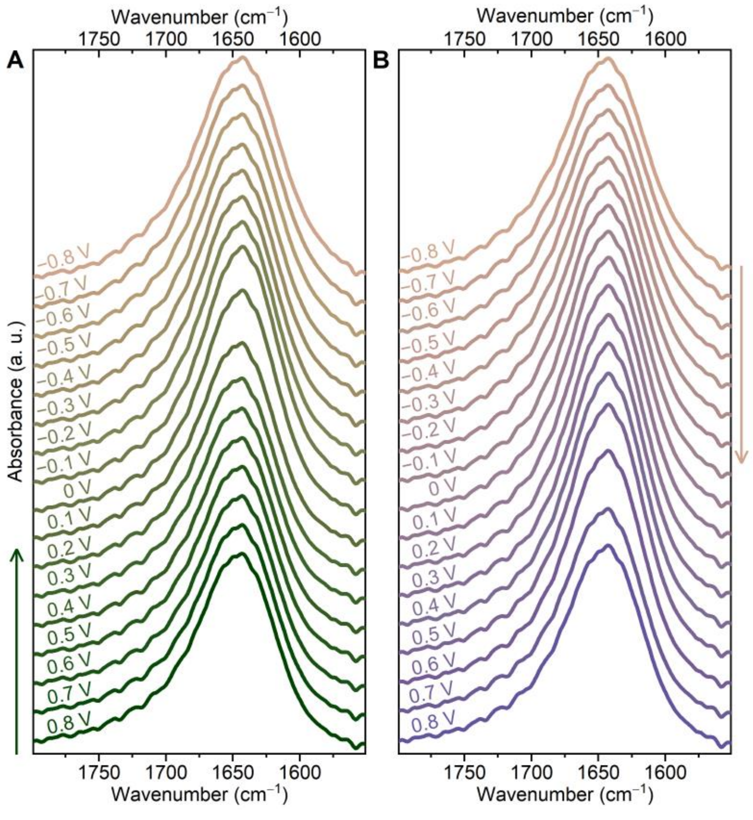

Figure S4. *Operando* FTIR spectra of $Ti_3C_2T_x$ MXene in 19.8 m LiCl recorded during (A) the negative-going scan from +0.8 V to −0.8 V (bottom to top) and (B) the positive-going scan from −0.8 V to 0.8 V (top to bottom). Arrows indicate scan direction.

Reproducibility: The infrared measurements show good stability of the $Ti_3C_2T_x$ MXene film (Figure S5). The infrared spectrum recorded at the end of a CV at +0.8 V is nearly identical to the spectrum recorded at the beginning of a CV at +0.8 V. The only difference is a small increase in bulk-like water that can be seen in the difference spectrum. For comparison, the difference spectrum at −0.8 V is also presented.

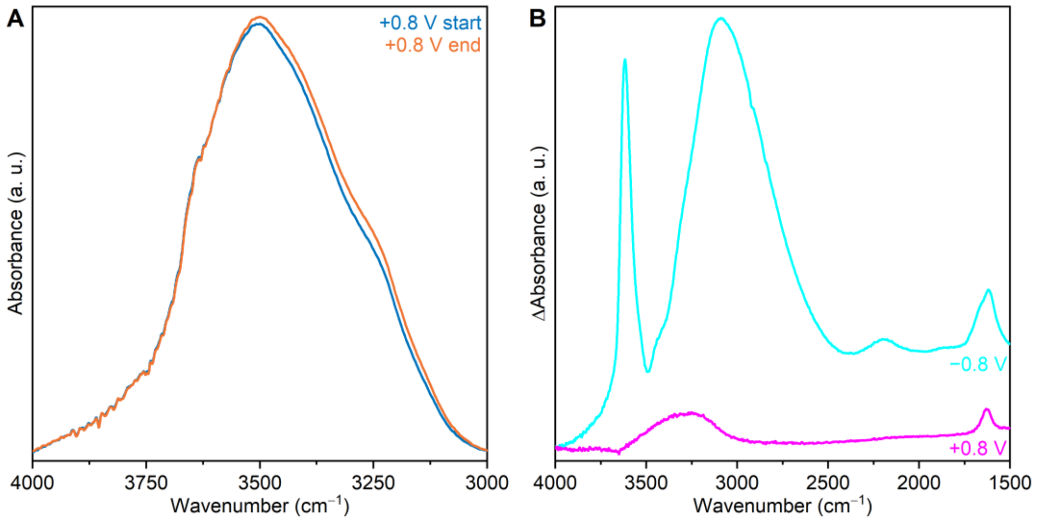

Figure S5. Reproducibility of IR measurements. (A) FTIR spectra at the start and end of a CV, both recorded at +0.8 V. (B) Difference spectra at −0.8 V and +0.8 V, spectrum at the start of the CV at +0.8 V subtracted.

Operando FTIR spectra in dilute electrolyte: Cyclic voltammetry performed in 0.1 M LiCl strongly differs from WiSE as shown in Figure S6. The potential window is smaller due to the low electrolyte concentration. For comparison, *operando* FTIR measurements were also performed over this potential window (Figure S7).

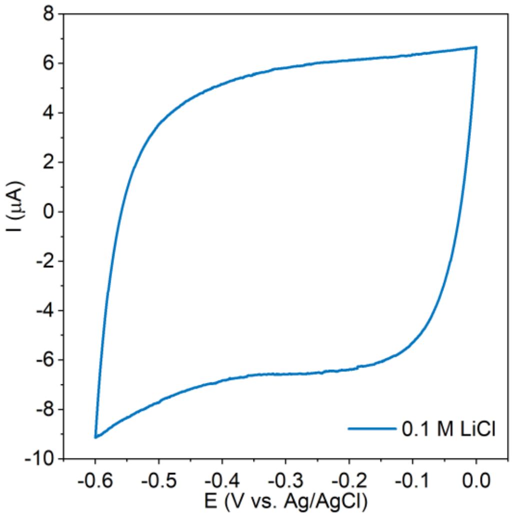

Figure S6. CV recorded during *operando* FTIR measurement of $Ti_3C_2T_x$ MXene electrode in 0.1 M LiCl.

The O-H stretch region was again fitted with the same three main components: 1, corresponding to the free O-H at ca. 3650 cm^{-1}; 2, representing weakly H-bonded water at ca. 3500 cm^{-1}; and 3, assigned to strongly H-bonded water at 3240 cm^{-1}. The frequencies of all three components redshift with increasingly negative potential. Component 4 at 3030 cm^{-1} appears at negative potentials, but doesn't exceed 3% of the total integrated area of the O-H stretch region. It is therefore not plotted in Figure S7A.

The spectral evolution in 0.1 M LiCl as a function of potential (Figure S7) is very similar to that observed in 0.1 M Li_2SO_4 (unpublished data, submitted), further corroborating that anions do not play a noticeable role in the charge storage mechanism in MXene. Peaks 1-3 (Figure S7A), corresponding to peaks 1-3 identified in concentrated electrolyte, are overlaid on the absolute spectra. In dilute electrolyte cations were found to intercalate in a partially desolvated form; the number of water molecules intercalating per Li^+ was determined to be between 1 and 1.5. (17-19) The negligible contribution from component 4 in dilute electrolyte (Figure S7D) is likely due to the much smaller number of Li^+ intercalated during the CV compared to concentrated LiCl. In WiSE we have approximately 0.25 Li^+ per surface Ti calculated from charge passed, whereas in 0.1 M LiCl that drops to 0.037 Li^+ per surface Ti. In addition to a smaller number of Li^+, we may also have more water in the interlayer space at dilute LiCl concentration due to osmotic swelling, which has been observed for clay materials (20) including $Ti_3C_2T_x$. (21) Also the MXene electrode is polarized to a much greater degree when the OCP is considered: in WiSE, the OCP was nearly +300 mV vs. Ag and the MXene electrode is polarized to −0.8 V. In 0.1 M LiCl the OCP was −200 mV vs. Ag/AgCl and the negative vertex potential is −0.6 V.

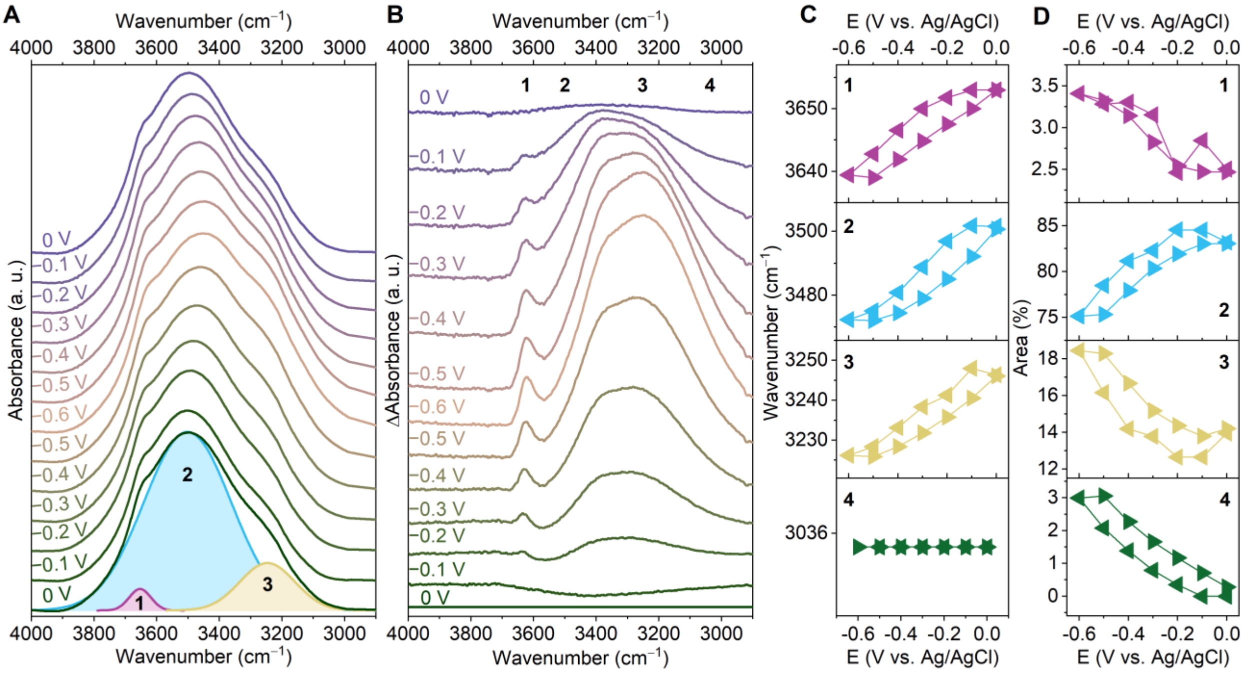

Figure S7. *Operando* FTIR spectra in dilute electrolyte. (A) Absorbance spectra of $Ti_3C_2T_x$ film in 0.1 M LiCl as a function of applied potential; shaded curves represent the peak fit of the initial spectrum at 0 V. (B) *Operando* FTIR data from panel (A) presented as difference spectra with the initial spectrum at 0 V subtracted. (C) Frequencies and (D) areas of the components from the peak fit as a function of applied potential.

REFERENCES

(1) Berdiyorov, G. R. Optical Properties of Functionalized $Ti_3C_2T_2$ (T = F, O, OH) MXene: First-Principles Calculations. *AIP Adv.* **2016**, *6* (5), 55105. https://doi.org/10.1063/1.4948799.

(2) Kulkarni, P.; Ghosh, D.; Balakrishna, R. G. Recent Progress in “Water-in-Salt” and “Water-in-Salt”-Hybrid-Electrolyte-Based High Voltage Rechargeable Batteries. *Sustain. Energy Fuels* **2021**, *5* (6), 1619–1654. https://doi.org/10.1039/D0SE01313G.

(3) Mason, P. E.; Ansell, S.; Neilson, G. W.; Rempe, S. B. Neutron Scattering Studies of the Hydration Structure of Li^+. *J. Phys. Chem. B* **2015**, *119* (5), 2003–2009. https://doi.org/10.1021/jp511508n.

(4) Egorov, A. V.; Komolkin, A. V.; Chizhik, V. I.; Yushmanov, P. V.; Lyubartsev, A. P.; Laaksonen, A. Temperature and Concentration Effects on Li^+-Ion Hydration. A Molecular Dynamics Simulation Study. *J. Phys. Chem. B* **2003**, 107, 14, 3234–3242. https://doi.org/10.1021/jp026677l.

(5) Lukatskaya, M. R.; Mashtalir, O.; Ren, C. E.; Dall’Agnese, Y.; Rozier, P.; Taberna, P. L.; Naguib, M.; Simon, P.; Barsoum, M. W.; Gogotsi, Y. Cation Intercalation and High Volumetric Capacitance of Two-Dimensional Titanium Carbide. *Science* **2013**, *341* (6153), 1502–1505. https://doi.org/10.1126/science.1241488.

(6) Shpigel, N.; Chakraborty, A.; Malchik, F.; Bergman, G.; Nimkar, A.; Gavriel, B.; Turgeman, M.; Hong, C. N.; Lukatskaya, M. R.; Levi, M. D.; Gogotsi, Y.; Major, D. T.; Aurbach, D. Can Anions Be Inserted into MXene? *J. Am. Chem. Soc.* **2021**, *143* (32), 12552–12559. https://doi.org/10.1021/jacs.1c03840.

(7) Ni, Y.; Skinner, J. L. IR and SFG Vibrational Spectroscopy of the Water Bend in the Bulk Liquid and at the Liquid-Vapor Interface, Respectively. *J. Chem. Phys.* **2015**, *143* (1), 14502. https://doi.org/10.1063/1.4923462.

(8) Riemenschneider, J.; Holzmann, J.; Ludwig, R. Salt Effects on the Structure of Water Probed by Attenuated Total Reflection Infrared Spectroscopy and Molecular Dynamics Simulations. *ChemPhysChem* **2008**, *9* (18), 2731–2736. https://doi.org/10.1002/cphc.200800571.

(9) Wei, Z.-F.; Zhang, Y.-H.; Zhao, L.-J.; Liu, J.-H.; Li, X.-H. Observation of the First Hydration Layer of Isolated Cations and Anions through the FTIR-ATR Difference Spectra. *J. Phys. Chem. A* **2005**, *109* (7), 1337–1342. https://doi.org/10.1021/jp0453865.
(10) Sun, Q. The Single Donator-Single Acceptor Hydrogen Bonding Structure in Water Probed by Raman Spectroscopy. *J. Chem. Phys.* **2010**, *132*, 054507. https://doi.org/10.1063/1.3308496.
(11) Sun, Q. Local Statistical Interpretation for Water Structure. *Chem. Phys. Lett.* **2013**, *568-569*, 90-94. https://doi.org/10.1016/j.cplett.2013.03.065.
(12) Ohno, K.; Okimura, M.; Akai, N.; Katsumoto, Y. The Effect of Cooperative Hydrogen Bonding on the OH Stretching-Band Shift for Water Clusters Studied by Matrix-Isolation Infrared Spectroscopy and Density Functional Theory. *Phys. Chem. Chem. Phys*. **2005**, *7*, 3005-3014. https://doi.org/10.1039/B506641G.
(13) Schmidt, D. A.; Miki, K. Structural Correlations in Liquid Water: A New Interpretation of IR Spectroscopy. *J. Phys. Chem. A* **2007**, 111, *40*, 10119-10122. https://doi.org/10.1021/jp074737n.
(14) Vinaykin, M.; Benderskii, A. V. Vibrational Sum-Frequency Spectrum of the Water Bend at the Air/Water Interface. *J. Phys. Chem Lett.* **2012**, *3* (22), 3348–3352. https://doi.org/10.1021/jz3014776.
(15) Hatch, C. D.; Wiese, J. S.; Crane, C. C.; Harris, K. J.; Kloss, H. G.; Baltrusaitis, J. Water Adsorption on Clay Minerals As a Function of Relative Humidity: Application of BET and Freundlich Adsorption Models. *Langmuir* **2012**, *28* (3), 1790–1803. https://doi.org/10.1021/la2042873.
(16) Falk, M. The Frequency of the H-O-H Bending Fundamental in Solids and Liquids. *Spectrochim. Acta A-M* **1984**, *40* (1), 43–48. https://doi.org/10.1016/0584-8539(84)80027-6.
(17) Wang, X.; Mathis, T. S.; Sun, Y.; Tsai, W.-Y.; Shpigel, N.; Shao, H.; Zhang, D.; Hantanasirisakul, K.; Malchik, F.; Balke, N.; Jiang, D.; Simon, P.; Gogotsi, Y. Titanium Carbide MXene Shows an Electrochemical Anomaly in Water-in-Salt Electrolytes. *ACS Nano* **2021**, *15* (9), 15274–15284. https://doi.org/10.1021/acsnano.1c06027.
(18) Shpigel, N.; Levi, M. D.; Sigalov, S.; Mathis, T. S.; Gogotsi, Y.; Aurbach, D. Direct Assessment of Nanoconfined Water in 2D Ti_3C_2 Electrode Interspaces by a Surface Acoustic Technique. *J. Am. Chem. Soc.* **2018**, *140* (28), 8910–8917. https://doi.org/10.1021/jacs.8b04862.
(19) Shpigel, N.; Lukatskaya, M. R.; Sigalov, S.; Ren, C. E.; Nayak, P.; Levi, M. D.; Daikhin, L.; Aurbach, D.; Gogotsi, Y. In Situ Monitoring of Gravimetric and Viscoelastic Changes in 2D Intercalation Electrodes. *ACS Energy Lett.* **2017**, *2* (6), 1407–1415. https://doi.org/10.1021/acsenergylett.7b00133.
(20) Norrish, K. Crystalline Swelling of Montmorillonite: Manner of Swelling of Montmorillonite. *Nature* **1954**, *173* (4397), 256–257. https://doi.org/10.1038/173256a0.
(21) Natu, V.; Pai, R.; Wilson, O.; Gadasu, E.; Badr, H.; Karmakar, A.; Magenau, A. J. D.; Kalra, V.; Barsoum, M. W. Effect of Base/Nucleophile Treatment on Interlayer Ion Intercalation, Surface Terminations, and Osmotic Swelling of $Ti_3C_2T_z$ MXene Multilayers. *Chem. Mater.* **2022**, *34* (2), 678–693. https://doi.org/10.1021/acs.chemmater.1c03390.
